# Aberrant Resting-State Functional Brain Connectivity of Insular Subregions in Obstructive Sleep Apnea

**DOI:** 10.3389/fnins.2021.765775

**Published:** 2022-01-20

**Authors:** Linghong Kong, Haijun Li, Yongqiang Shu, Xiang Liu, Panmei Li, Kunyao Li, Wei Xie, Yaping Zeng, Dechang Peng

**Affiliations:** Department of Radiology, The First Affiliated Hospital of Nanchang University, Nanchang, China

**Keywords:** insula, functional connectivity, brain network, cognitive impairment, magnetic resonance imaging

## Abstract

The insular cortex is a cortical regulatory area involved in dyspnea, cognition, emotion, and sensorimotor function. Previous studies reported that obstructive sleep apnea (OSA) shows insular tissue damage and abnormal functional connections for the whole insula. The insula can be divided into different subregions with distinct functional profiles, including the ventral anterior insula (vAI) participating in affective processing, dorsal anterior insula (dAI) involved in cognitive processing, and posterior insula (PI) involved in the processing of sensorimotor information. However, the functional connectivity (FC) of these insular subregions in OSA has yet to be established. Hence, the purpose of this study was to explore the resting-state FC of the insular subregions with other brain areas and its relationship with clinical symptoms of OSA. Resting-state functional magnetic resonance imaging data from 83 male OSA patients and 84 healthy controls were analyzed by whole-brain voxel-based FC using spherical seeds from six insular subregions, namely, the bilateral vAI, dAI, and PI, to identify abnormalities in the insular subregions network and related brain regions. Ultimately, the Pearson correlation analysis was carried out between the dysfunction results and the neuropsychological tests. Compared with the healthy control group, the OSA patients exhibited disturbed FC from the dAI to areas relevant to cognition, such as the bilateral cerebellum posterior lobe, superior frontal gyrus, right middle frontal gyrus and middle temporal gyrus; decreased FC from the vAI to areas linked with emotion, such as the bilateral fusiform gyrus, superior parietal lobule, precuneus and cerebellum posterior lobe; and abnormal FC from the PI to the brain regions involved in sensorimotor such as the bilateral precentral gyrus, right superior/middle temporal gyrus and left superior frontal gyrus. The linear regression result showed that the apnea-hypopnea index was positively correlated with the increased FC between the right PI and the right precuneus (after Bonferroni correlation, *P* < 0.001) In conclusion, the abnormal FC between insular subregions and other brain regions were related to cognitive, emotional and sensorimotor networks in OSA patients. These results may provide a new imaging perspective for further understanding of OSA-related cognitive and affective disorders.

## Introduction

Obstructive sleep apnea (OSA) is a sleep-related breathing disorder characterized by recurrent complete or partial pharyngeal collapse during sleep (Jordan et al., 2014). These episodes bring about intermittent nocturnal apnea and hypoxia, hypercapnia, and sleep fragmentation, negatively impacting cognitive, neuropsychological, sensorimotor, and affective functioning (Vanek et al., 2020; Beaudin et al., 2021; Lee et al., 2021). Evidence has displayed that the occurrence of OSA is related to abnormal structure and function in the central nervous system, especially impairments in the insular cortex (Li et al., 2014; Rosenzweig et al., 2014). However, why is the insula stressed in the progression of OSA?

The human insular cortex, which accounts for less than 2% of the total cortical surface area, is extensively connected with the frontal, temporal, cerebellar and limbic regions (Ghaziri et al., 2017) and is implicated in a large number of widely different functions, ranging from cognition and speech production to the processing of emotions (Nieuwenhuys, 2012), all of which, coincidentally, are important characteristics in OSA. An increasing number of neuroimaging studies have indicated that prominent activation of the insular cortex is involved in sleep-disordered breathing (von Leupoldt and Dahme, 2005; Davis et al., 2008; Schon et al., 2008), which occurs in OSA due to airway collapse. Specifically, activation of the insular cortex increases the control of the habenula on the raphe nucleus, reducing the release of 5-hydroxytryptamine, decreasing muscle tension of the genioglossus and blocking the airway (Wang et al., 2014; Li et al., 2015). A meta-analysis highlighted the importance of the insular cortex in somatosensory, neurocognitive, perceptual, and affective deficits in OSA subjects (Tahmasian et al., 2016). Forasmuch, insular cortex, a multimodal integration region, plays a crucial role in the development of OSA, which is in line with the perspective of Cui et al. (2012). Damage to the insula may be related to cognitive, neuropsychological, sensorimotor and emotional impairments.

Interestingly, some research in animals and humans has demonstrated that the insular cortex is not homogeneous and that it can be functionally subdivided into meaningful subregions with different connectivity patterns (Kurth et al., 2010; Deen et al., 2011). The dorsal anterior insula (dAI) broadly maps onto cognitive tasks involving attention, memory, speech, and executive control. The ventral anterior insula (vAI) is associated with emotion, chemosensation, and autonomic function, and the posterior insula (PI) is linked primarily to pain, sensorimotor function and language, according to previous studies (Chang et al., 2013; Uddin et al., 2014). Therefore, anomalies in different insular subregions may result in impaired local functional magnetic resonance imaging (fMRI) responses to evoked cognitive function, emotions, sensorimotor function and dyspnea. In fact, abnormalities in the insula have been revealed in studies of brain functional activity in OSA subjects. For example, Peng et al. (2014) found that higher regional homogeneity (ReHo) occurred in the insula of male patients with severe OSA. In addition, altered limbic-striatal-cortical functional connectivity was reported in studies of altered hippocampal resting-state functional networks in OSA (Song et al., 2018; Kang et al., 2020; Zhou et al., 2020). Although ReHo and resting-state functional connectivity (FC) are both based on resting-state fMRI, ReHo, a method for analyzing the coherence and similarities of intraregional spontaneous low-frequency (0.01–0.08 Hz) signal fluctuations was used to detect unpredicted hemodynamic responses (Zang et al., 2004). Resting-state FC, focusing on the statistical correlation between signals in the whole brain or regional brain areas (Fox and Raichle, 2007), has shown substantial clinical research potential for exploring synchronous brain activity, which is also the highlight of the current research. Region-of-interest (ROI) studies based on resting-state FC have been considered sensitive and reliable (Hampson et al., 2002), and these techniques have been widely applied in various objective analyses of brain functional activity, varying from sleep disorders to neuropsychiatric diseases (Lei et al., 2019; Guo et al., 2020). Thus, resting-state FC studies based on ROI may be incredibly useful in evaluating insular FC integrity in patients with OSA. Nevertheless, the insula is rarely regarded as an ROI in studies of resting-state FC in OSA; A small number of studies that have focused on the insular cortex (Zhang et al., 2015; Park et al., 2016a), regarded it as a whole. The characteristics of rs-FC between different subregions of the insula and all other brain regions and the neural mechanisms underlying OSA remain unclear.

Consequently, based on the above reasons, we hypothesized that FC integrity in insular subregions would be impaired in OSA subjects in brain regions that regulate affective, cognitive and sensorimotor function. To test this hypothesis, we first investigated the rs-FC patterns of insular subregions with other brain regions using spherical seed ROI-based resting-state FC analysis in newly diagnosed untreated OSA participants and healthy controls (HCs) and then explored the differences in functional connectivity of insular subregions with other brain regions across the whole brain between the two groups. Subsequently, we evaluated possible correlations between aberrant rs-FC changes in insular subregions and OSA severity as well as neuropsychological performance to analyze the potential neuroimaging process of cognitive deficits in OSA patients.

## Materials and Methods

### Subjects

Ninety-two newly diagnosed untreated male patients with OSA and eighty-nine male HCs matched in age and education were enrolled in this study. All patients were enrolled from the Sleep Monitoring Room at the Respiratory Department of the First Affiliated Hospital of Nanchang University. HCs were recruited through local advertisements. Patients were jointly diagnosed by 3 experienced respiratory doctors based on the criteria from the Clinical Practice Guideline for Diagnostic Testing for Adult Obstructive Sleep Apnea proposed by the American Academy of Sleep Medicine (AASM) in 2017 (Kapur et al., 2017). Patients with moderate to severe OSA, indicated by an apnea-hypopnea index (AHI) ≥15, were selected for this study. All participants were males, right-handed, native Chinese speakers, aged from 22 to 60 years. The exclusion criteria for all subjects were as follows: (1) sleep disorders other than OSA; (2) history of cardiovascular diseases, respiratory diseases, diabetes, hypothyroidism or central nervous system disorders that included neurodegenerative disease, epilepsy, depression, tumor, brain trauma or infarction; (3) abuse of alcohol or illicit drugs or current intake of psychoactive medications; (4) MRI contraindications, such as claustrophobia, metallic implants, or devices in the body; and (5) suboptimal imaging data quality. The HCs did not have a current or lifetime history of sleep-related disorders or a history of psychotic, mood, or sleep-related disorders in first-degree relatives. Finally, 83 patients with OSA and 84 HCs were included. This study was approved by the Medical Research Ethics Committee of The First Affiliated Hospital of Nanchang University, and each participant provided written informed consent.

### Polysomnography and Neuropsychological Measures

To make an accurate diagnosis while excluding sleep breathing disorders other than OSA or potential sleep disturbances in HCs, all HCs and OSA subjects were required to undergo overnight polysomnography (PSG) using a Respironics LE-Series physiological monitoring system (Alice 5 LE, Respironics, Orlando, FL, United States). The day before PSG, all participants were asked to avoid hypnotics, alcoholic beverages and coffee. The PSG monitor assessed standard electroencephalogram (EEG) derivations from frontal, central, and occipital regions, F4/M1, C4/M1, and O2/M1, and back-up derivations, F3/M2, C3/M2, and O1/M2; electrooculograms (EOGs) obtained from the cornea and retina; chin electromyogram (EMG) obtained from three chin electrodes and one placed on the middle of the right anterior tibialis; electrocardiogram (ECG); body position; thoracic and abdominal respiratory movements; oral and nasal airflow; snoring; and oxygen saturation (SaO_2_). Total sleep time, sleep latency, sleep efficiency, sleep stages, arousal, and respiratory events were likewise recorded. PSG was conducted from 10 PM to 6 AM the next morning.

Obstructive apnea was described as a continuous reduction in airflow ≥90% for ≥10 s along with evident respiratory effort. Hypopnea was identified as a 30% or greater drop in airflow for ≥10 s accompanied by ≥4% oxygen desaturation or alternatively, a 50% or greater drop in airflow lasting ≥10 s accompanied by ≥3% oxygen desaturation or EEG arousal. The AHI was obtained from the aggregate numbers of apnea and hypopnea events per hour during sleep. According to the American Academy of Sleep Medicine Clinical Practice Guideline for Adult Obstructive Sleep Apnea (Berry et al., 2012, 2017), an AHI from 5 to 15 was diagnosed as mild; an AHI ≥ 15/h but <30/h was considered moderate, and an AHI ≥ 30/h was considered severe OSA.

The Epworth Sleepiness Scale (ESS) assess contains eight different categories and was used to evaluate self-reported answers to sleep questions regarding daytime sleepiness in OSA and control subjects. ESS total scores can range from 0 to 24 (each category scored from 0 to 3), and scores of 0, 6, 11, and 16 correspond to four different sleepiness levels (Johns, 1991). The Montreal Cognitive Assessment (MoCA), assessing executive functions, orientation, abstraction, attention, calculation, conceptual thinking, language, and memory, was used to assess cognitive function. A total MoCA score lower than 26 indicated cognitive impairment (Chen et al., 2016).

### Magnetic Resonance Imaging Data Acquisition

All images were captured on a 3.0 Tesla MRI system with an 8-channel phased-array head coil (Siemens, Munich, Germany). To reduce patient head movement and minimize scanner noise, we used foam pads and earplugs. First, conventional T1-weighted imaging (repetition time (TR) = 250 ms, echo time (TE) = 2.46 ms, thickness = 5 mm, gap = 1.5 mm, field-of-view (FOV) = 220 mm × 220 mm, slices = 19) and T2-weighted imaging (TR = 4,000 ms, TE = 113 ms, thickness = 5 mm, gap = 1.5 mm, FOV = 220 mm × 220 mm, slices = 19) were performed. rs-fMRI data were collected using a Gradient-Recalled Echo-planar Imaging (EPI) pulse sequence with the following parameters: TR = 2,000 ms, TE = 30 ms, flip angle = 90°, FOV = 230 mm × 230 mm, matrix = 64, thickness = 4 mm, gap = 1.2 mm. Each brain volume consisted of 30 axial sections, and each functional run comprised 240 volumes. During the rs-fMRI scans, all subjects were asked to close their eyes, stay as still as possible, not think about anything specific, and not fall asleep. Finally, high-resolution three-dimensional T1-weighted images were obtained by using a spoiled gradient-recalled echo sequence (TR = 1,900 ms, TE = 2.26 ms, thickness = 1.0 mm, gap = 0.5 mm, FOV = 250 mm × 250 mm, matrix = 256 × 256, flip angle = 9°, 176 sagittal slices). The images were read jointly by two senior radiologists to rule out obvious brain lesions.

### Functional Magnetic Resonance Imaging Data Preprocessing

MRIcro software^1^ was used to check the resting-state fMRI data and discard suboptimal ones. resting-state fMRI data were preprocessed with the Data Processing & Analysis for Brain Imaging toolbox DPABI^2^ (Yan et al., 2016), which was run on MATLAB 2018b (Mathworks, Natick, MA, United States). First, the first 10 time points were removed. Then the remaining 230 volumes were slice-time corrected and three-dimensional head-motion rectified. Three OSA patients and two HCs were excluded because the maximum displacement in any direction (*x*, *y*, or *z*) was more than 2.0 mm, or the angular rotation on any axis exceeded 2.0°, or the framewise displacement (FD) a relative displacement measure (Power et al., 2012) of any of the 230 volumes exceeded 2.5 standard deviations during the entire fMRI scanning process. Then, all the functional data were spatially normalized to the Montreal Neurological Institute (MNI) template using non-linear transformation procedures with SPM12 (Pirzada et al., 2020), resampled to 3 mm × 3 mm × 3 mm voxels, and smoothed with a 6-mm full-width at half-maximum (FWHM) filter. Finally, a temporal filter (0.01–0.08 Hz) was utilized to suppress the effects of low-frequency drift and high-frequency noise. To further reduce the influence of confounding factors, a multiple regression method was performed to regress interference signals, including the mean time series of all voxels from white matter, cerebrospinal fluid, global signals and Friston 24-parameters head motion (6 head motion parameters, 6 head motion parameters one time point before, and the 12 corresponding squared items) (Friston et al., 1996; Saad et al., 2012; Satterthwaite et al., 2013).

### Seed Definition and Functional Connectivity Analysis

The whole insula was divided into three different functional subregions based on previous cluster analysis of the resting-state FC (Kurth et al., 2010; Deen et al., 2011; Uddin et al., 2014). According to the previous study (Li et al., 2019), the six subregions of the bilateral insula were defined as spherical seed ROIs with a radius of 6 mm and the following MNI coordinates: left vAI (−33, 13, −7), right vAI (32, 10, −6), left dAI (−38, 6, 2), right dAI (35, 7, 3), left PI (−38, −6, 5), and right PI (35, −11, 6).

The functional connectivity with different subregions ROIs was calculated by DPABI (see text footnote 2). First, the time series of each spherical seed region was extracted, that is, the average value of the fMRI time series of all voxels in the region. For each seed region, the Pearson correlation coefficients of the time series with that of all other voxels in the brain were calculated. The resulting correlation matrix is the whole-brain FC map of each subject. Finally, the Fisher r-to-z transform was utilized to transform the correlation graph into a *z*-value map to improve the normality of the correlation coefficients.

### Statistical Analysis

The demographic and clinical data of the two groups were compared by independent sample *t*-tests with Statistical Package for the Social Sciences version 24.0 (SPSS, Chicago, IL, United States); when *p* < 0.05, the differences are considered significant.

For the rs-FC between each ROI and the remaining whole brain voxels, one-sample *t*-tests were analyzed in two groups to identify spatial distribution, respectively. Then, analysis of covariance was applied to analyze intergroup differences, with body mass index (BMI) and age as covariates using DPABI (see text footnote 2) software in MATLAB 2018b (Mathworks, Natick, MA, United States). The results were corrected using AlphaSim^3^ implemented in DPABI Viewer part with *p* < 0.001 and were reported using REST V1.8^4^ (Song et al., 2011). The resulting *z*-value maps were overlaid on the rendered views using BrainNet Viewer, and the anatomy of the surviving brain regions was reported using xjView software^5^.

To examine whether there is a relationship between the abnormal FC and clinical evaluation. First, we preserved the abnormal brain regions as masks. We then extract the functional connection signal *z*-value for each mask in every subject using REST V1.8 (see text footnote 4). Linear regression analysis was calculated between the averaged *z*-values of the abnormal brain regions and clinical variables, with BMI and age as covariates using SPSS version 24.0. *P* < 0.001 was considered to be statistically significant for each set of independent variables (10 clinical variables × 36 regions) after the Bonferroni correlation.

## Results

### Demographic and Clinical Characteristics

The demographic and clinical data from each group are summarized in Table 1. There were no significant differences in age, education years, total sleep time, stage 2 sleep or mean FD between the OSA patients and HCs (*P* > 0.05). Compared with the HC group, the OSA group had significantly higher BMI, AHI, stage 1 sleep, SaO_2_ <90%, AI and ESS scores and significantly lower sleep efficiency, stage 3 + 4 sleep, rapid eye movement REM sleep, nadir SaO_2_ and MoCA scores.

**TABLE 1:** Demographic and clinical data between OSA patients and HCs.

Characteristic	OSA patients (*N* = 83)		HCs (*N* = 84)		*t*-values	*P*-value
	Mean	SD	Mean	SD		
Age, years	40.7	10.2	42.4	12.1	−1.39	0.124
Education, years	12.5	3.6	11.8	3.3	0.92	0.342
BMI, kg/m^2^	26.8	3.5	22.6	2.2	7.13	< 0.001
Total sleep time, min	359.8	97.2	389.3	30.2	−2.35	0.012
Sleep efficiency, %	82.9	19.8	90.8	5.8	−4.15	< 0.001
AHI/hour	51.3	21.3	2.5	1.3	18.91	< 0.001
Stage 1, %	27.9	15.1	10	3.8	6.67	< 0.001
Stage 2, %	40.3	13.9	38.7	6.6	0.9	0.362
Stage 3 + 4, %	20.2	15.8	24.2	7.9	−3.89	< 0.001
REM, %	11.3	10.2	21.3	7.7	−7.58	< 0.001
SaO_2_ <90%	23.4	18.7	0.2	0.2	13.45	< 0.001
Nadir SaO_2_, %	70.3	12.2	92.6	4.72	−10.56	< 0.001
AI/hour	47.0	25.4	10.5	3.1	7.96	< 0.001
ESS, score	10.0	4.6	3.4	2.2	10.5	< 0.001
MoCA, score	24.9	3.2	28.6	1.4	−6.53	< 0.001
Mean FD, mm	0.068	0.036	0.059	0.031	1.73	0.08

*OSA, obstructive sleep apnea; HCs, healthy controls; N, number; SD, standard deviation; BMI, body mass index; AHI, apnea-hypopnea index; REM, rapid eye movement; SaO_2_, oxygen saturation; AI, arousal index; SaO_2_ <90%, percentage of total sleep time spent at oxygen saturation less than 90%; ESS, Epworth Sleepiness Scale; MoCA, Montreal Cognitive Assessment; FD, framewise displacement.*

### Resting-State Functional Connectivity Patterns Based on Seed Regions of Interest

**FIGURE 1:**
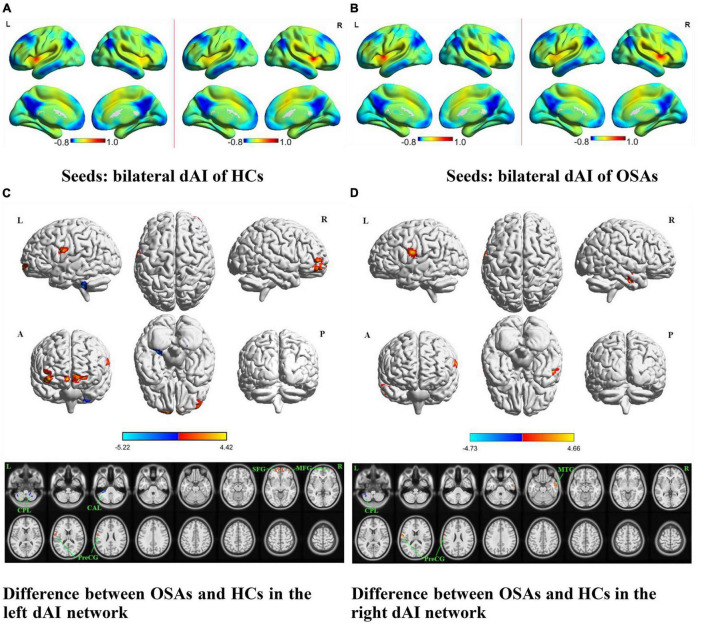
The spatial distribution of the FC maps of the bilateral dAI in the two groups and between-group comparisons of the rs-FC of the bilateral dAI. The FC patterns of the bilateral dAI in the HC and OSA groups are shown in **(A,B)**, respectively. Color bars indicate *z*-values; warmer colors indicate higher *z*-values relative to the whole-brain average, while colder colors indicate lower *z*-values. **(C)** Denotes the altered FC based on the left dAI seed in OSAs relative to HCs. **(D)** Denotes the altered FC based on the right dAI seed in OSAs relative to HCs (voxel-level *P* < 0.001, AlphaSim corrected). Color bars indicate t-scores; warm colors indicate areas where the FC value in OSAs is greater than that in HCs, while cold colors indicate the opposite. HCs, healthy controls; OSAs, patients with obstructive sleep apnea; dAI, dorsal anterior insula; FC, functional connectivity; SFG, superior frontal gyrus; MFG, middle frontal gyrus; MTG, middle temporal gyrus; PreCG, precentral gyrus; CPL, cerebellum posterior lobe; CAL, cerebellum anterior lobe; L, left; R, right; A, anterior; P, posterior.

**FIGURE 2:**
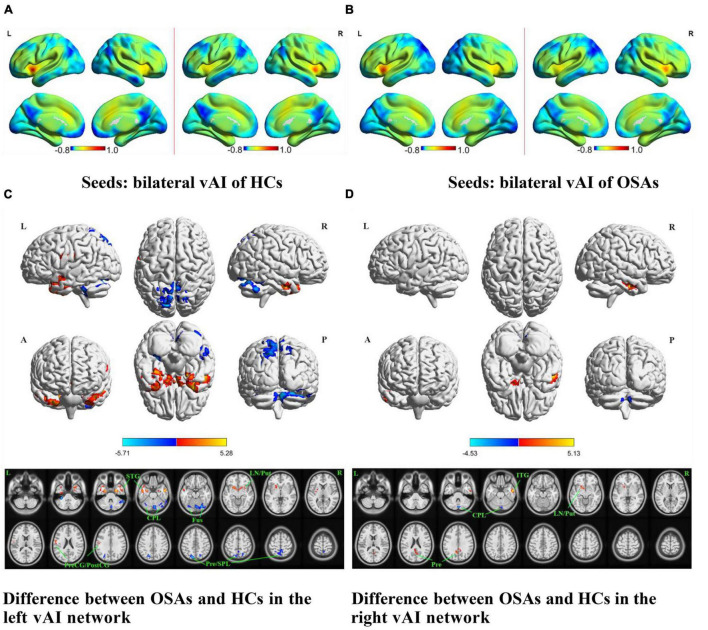
The spatial distribution of the FC maps of the bilateral vAI in the two groups and between-group comparisons of the rs-FC of the bilateral vAI. The FC patterns of the bilateral vAI in the HC and OSA groups are shown in **(A,B)**, respectively. Color bars indicate *z*-values; warmer colors indicate higher *z*-values relative to the whole-brain average, while colder colors indicate lower *z*-values. **(C)** Denotes the altered FC based on the left vAI seed in OSAs relative to HCs. **(D)** Denotes the altered FC based on the right vAI seed in OSAs relative to HCs (voxel-level *P* < 0.001, AlphaSim corrected). Color bars indicate t-scores; warm colors indicate areas where the FC value in OSAs is greater than that in HCs, while cold colors indicate the opposite. HCs, healthy controls; OSAs, obstructive sleep apnea patients; vAI, ventral anterior insula; FC, functional connectivity; STG, superior temporal gyrus; ITG, inferior temporal gyrus; LN, lentiform nucleus; Put, putamen; PreCG, precentral gyrus; PostCG, postcentral gyrus; CPL, cerebellum posterior lobe; Fus, fusiform gyrus; SPL, superior parietal lobule; Pre, precuneus; L, left; R, right; A, anterior; P, posterior.

**FIGURE 3:**
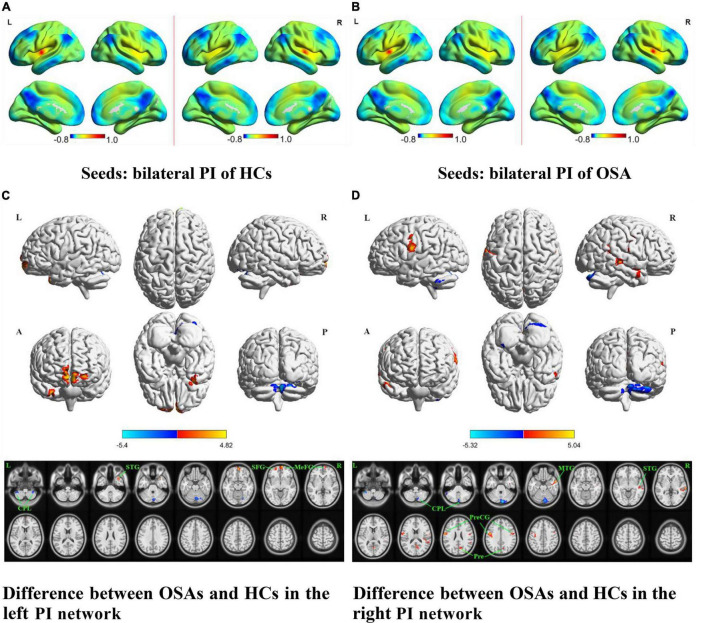
The spatial distribution of the FC maps of the bilateral PI in the two groups and between-group comparisons of the rs-FC of the bilateral PI. The FC patterns of the bilateral PI in the HC and OSA groups are shown in **(A,B)**, respectively. Color bars indicate *z*-values; warmer colors indicate higher *z*-values relative to the whole-brain average, while colder colors indicate lower *z*-values. **(C)** Denotes the altered FC based on the left PI seed in OSAs relative to HCs. **(D)** Denotes the altered FC based on the right PI seed in OSAs relative to HCs (voxel-level *P* < 0.001, AlphaSim corrected). Color bars indicate t-scores; warm colors indicate areas where the FC value in OSAs is greater than that in HCs, while cold colors indicate the opposite. HCs, healthy controls; OSAs, obstructive sleep apnea patients; PI, posterior insula; FC, functional connectivity; SFG, superior frontal gyrus; MeFG, medial frontal gyrus; STG, superior temporal gyrus; MTG, middle temporal gyrus; PreCG, precentral gyrus; Pre, precuneus; CPL, cerebellum posterior lobe; L, left; R, right; A, anterior; P, posterior.

The OSA patients and HCs showed similar resting-state functional connectivity patterns for the different insular functional subregions, as shown in Figures 1A,B, 2A,B, 3A,B.

### Differences in Resting-State Functional Connectivity Results Between the Two Groups

The results of intergroup rs-FC comparisons at the voxel-based whole-brain level based on bilateral dAI spherical seed points are displayed in Figure 1 and Table 2. When taking the left dAI as the seed, the patients with OSA showed significantly lower rs-FC with the bilateral cerebellum posterior lobe (CPL) and left cerebellum anterior lobe but significantly higher rs-FC with the bilateral superior frontal gyrus (SFG), right middle frontal gyrus (MFG), and left precentral gyrus (PreCG) than the HCs (Figure 1C). When taking the right dAI as the seed, the patients with OSA showed significantly lower rs-FC with the left CPL but significantly higher rs-FC with the right middle temporal gyrus (MTG) and left PreCG than the HCs (Figure 1D).

**TABLE 2:** Brain areas showing functional connectivity differences with insular subdivisions between OSA patients and HCs.

Seed-ROIs	Brain areas	L/R	MNI coordinates		Num. of voxels		*T*-values
			*x*	*y*	*z*		
**Left dAI**							
	Cerebellum posterior lobe	L	−27	−51	−60	49	–5.225
	Cerebellum posterior lobe	R	18	−42	−57	40	–4.172
	Cerebellum anterior lobe	L	−30	−36	−42	37	–4.971
	Middle frontal gyrus	R	42	60	−6	46	4.016
	Superior frontal gyrus	R	12	54	−9	33	4.266
	Superior frontal gyrus	L	−6	66	−6	39	4.423
	Precentral gyrus	L	−60	−3	21	43	4.058
**Right dAI**							
	Cerebellum posterior lobe	L	−27	−51	−63	47	−4726
	Middle temporal gyrus	R	54	−3	−24	49	4.422
	Precentral gyrus	L	−63	−6	18	49	4.661
**Left vAI**							
	Cerebellum posterior lobe	L	−30	−36	−39	41	–5.450
	Cerebellum posterior lobe, fusiform gyrus	R	27	−75	−18	421	–5.485
	Fusiform gyrus	L	−33	−72	−21	62	–4.521
	Precuneus, superior parietal lobule	L	−12	−78	45	298	–5.713
	Precuneus, superior parietal lobule	R	18	−69	48	51	–4.322
	Superior temporal gyrus	L	−27	−6	−33	239	4.837
	Superior temporal gyrus	R	24	−3	−30	237	5.275
	Lentiform nucleus, putamen	R	6	−3	−15	158	4.601
	Precentral/Postcentral gyrus	L	−54	−15	24	62	4.187
**Right vAI**							
	Cerebellum posterior lobe	R	0	−78	−33	38	–4.529
	Inferior temporal gyrus	R	54	−9	−27	74	5.127
	Lentiform nucleus, putamen	L	−18	9	−12	52	4.359
	Precuneus	L	−6	−57	27	97	4.110
**Left PI**							
	Cerebellum posterior lobe	L	−30	−45	−60	70	–4.815
	Cerebellum posterior lobe	R	6	−72	−24	129	–4.437
	Superior temporal gyrus	R	33	21	−36	31	4.629
	Medial frontal gyrus	R	9	69	−6	136	4.824
	Superior frontal gyrus	L	−21	60	−6	30	4.071
**Right PI**							
	Cerebellum posterior lobe	L	−36	−45	−54	72	–5.024
	Cerebellum posterior lobe	R	6	−87	−27	246	–5.321
	Middle temporal gyrus	R	54	3	−21	104	5.040
	Precuneus	R	3	−60	18	97	4.337
	Superior temporal gyrus	R	45	−33	12	68	4.111
	Precentral gyrus	L	−48	−3	36	207	4.695
	Precentral gyrus	R	48	0	33	55	4.287

*Voxel level P < 0.001, AlphaSim corrected. OSA, obstructive sleep apnea; HCs, healthy controls; dAI, dorsal anterior insula; vAI, ventral anterior insula; PI, posterior insula; L, left; R, right.*

The results of intergroup rs-FC comparisons at the voxel-based whole-brain level based on bilateral vAI seed points are shown in Figure 2 and Table 2. When taking the left vAI as the seed, the patients with OSA had significantly lower rs-FC with the bilateral CPL, fusiform gyrus, superior parietal lobule, and precuneus but significantly higher rs-FC with the bilateral superior temporal gyrus (STG), right lentiform nucleus and putamen, and left PreCG and postcentral gyrus than the HCs (Figure 2C). When taking the right vAI as the seed, the patients with OSA showed significantly lower rs-FC with the right CPL but significantly higher rs-FC with the right inferior temporal gyrus, left lentiform nucleus, putamen and precuneus than the HCs (Figure 2D).

The results of intergroup rs-FC comparisons at the voxel-based whole-brain level based on bilateral PI seed points are displayed in Figure 3 and Table 2. When taking the left PI as the seed, the patients with OSA showed significantly lower rs-FC with the bilateral CPL but significantly higher rs-FC with the right STG and MFG and left SFG than the HCs (Figure 3C). When taking the right PI as the seed, the patients with OSA showed significantly lower rs-FC with the bilateral CPL but significantly higher rs-FC with the bilateral PreCG and right MTG, STG, and precuneus than the HCs (Figure 3D).

### Correlations Between rs-FC Showing Group Differences and Clinical Variables in Obstructive Sleep Apnea Patients

To explore symptom-related insular functional connectivity, linear correlation analysis between rs-FC and clinical variables was performed. Table 3 exhibited statistically significant results with *P* < 0.05 before multiple comparison correction. However, only the AHI was positively correlated with the increased FC between the right PI and the right precuneus after Bonferroni correlation with *P* < 0.001 among the OSA patients. There was no significant correlation between FC values and the clinical and neuropsychologic variables in the control group.

**TABLE 3:** Significant associations between insular functional connectivity and clinical characteristic in OSA patients.

FC between brain regions	Clinical characteristic	*t*-values	*P*-values
Left dAI-left precentral gyrus	AHI	2.625	0.01
Left dAI-right superior frontal gyrus	AHI	2.009	0.048
Right dAI-left precentral gyrus	AHI	2.479	0.015
Left vAI-left cerebellum posterior lobe	AHI	–2.424	0.018
Right PI-right cerebellum posterior lobe	AHI	–2.497	0.015
Right PI-right precuneus	AHI	3.954	< 0.001**
Right PI-right superior temporal gyrus	AHI	3.036	0.003
Right PI-right precentral gyrus	AHI	2.466	0.016
Left dAI-right cerebellum posterior lobe	AI	–2.027	0.046
Left dAI-left cerebellum anterior lobe	AI	–2.246	0.028
Left vAI-left cerebellum posterior lobe	AI	–2.861	0.005
Left vAI-left precuneus, superior parietal lobule	AI	–2.228	0.029
Right PI-right middle temporal gyrus	AI	2.383	0.02
Right PI-right precuneus	AI	2.301	0.024
Right PI-right superior temporal gyrus	AI	2.730	0.008
Right PI-left precentral gyrus	AI	2.479	0.015
Left dAI-right superior frontal gyrus	Nadir SaO_2_	–2.207	0.03
Right PI-right precuneus	Nadir SaO_2_	–2.655	0.01
Left dAI-right superior frontal gyrus	MoCA	–2.051	0.043
Left dAI-left superior frontal gyrus	MoCA	–2.344	0.022
Left vAI-left fusiform gyrus	ESS	–2.096	0.039
Left vAI-right precuneus, superior parietal lobule	Sleep efficiency	2.236	0.028
Right vAI-right inferior temporal gyrus	Sleep efficiency	–2.703	0.008
Left vAI-right lentiform nucleus, putamen	Stage 1	2.143	0.035
Left dAI-left superior frontal gyrus	REM	–2.291	0.025
Left PI-left superior frontal gyrus	REM	–3.283	0.002

**Exist statistical difference after Bonferroni correction. OSA, obstructive sleep apnea; HCs, healthy controls; vAI, ventral anterior insula; dAI, dorsal anterior insula; PI, posterior insula; AHI, apnea-hypopnea index; ESS, Epworth Sleepiness Scale; AI, arousal index; MoCA, Montreal Cognitive Assessment; SaO_2_, oxygen saturation; REM, rapid eye movement.

## Discussion

To the best of our knowledge, this study is the first to explore the FC of insular subregions using spherical seed functional connectivity analysis in OSA patients. Our results showed that abnormal functional connections of the vAI, dAI, and PI were distributed across different brain regions separately involving emotion, cognition (executive function, attention, and memory) and sensorimotor function. Structural or functional damage has also been shown in these brain regions in previous studies on OSA, and the abnormal functional connections in these areas were related to sleep and neuropsychological variables, which is consistent with our hypothesis. Additionally, we found that there were differential FC patterns between the two sides of the insula, which was similar to previous laterality results observed in this part of the brain (Van Essen et al., 2012), which may be due to the distinct order of bilateral insular lobe development: the surface area of the left lobe is larger than that of the right lobe, and the right lobe stops growing earlier than the left lobe (Cauda et al., 2011). In addition, resting-state brain activity may be affected by obesity, age and sex, the main risk factors for OSA, as shown in previous studies (Black et al., 2014; Li et al., 2016; Kumar et al., 2021). In our study, we also found that BMI in the OSA patients was significantly higher than that in HCs. To avoid the influence of the above three factors on the rs-FC results between OSA and HC patients, we recruited only adult male OSA patients and used BMI and age as covariates.

### Deficits in Functional Connectivity Between the Dorsal Anterior Insula and Cognitive Networks

In the current study, we found that compared with HCs, OSA patients showed abnormal FC between the dAI and certain cognitive regulatory brain regions, including the CPL, SFG, MFG, and MTG. The dAI is considered an influential functional center of the human brain and is often described as a “cognitive” area (Uddin et al., 2014). Growing evidence suggests that respiratory and sleep disorders in OSA patients lead to cognitive impairment, encompassing language, attention, executive control and so on (Beaudin et al., 2021). Newly emerging evidence has demonstrated that damage to the cerebellar cortex, including among abnormally connected brain regions, leads to cognitive impairment (Ramnani, 2012). The CPL is associated with cognitive processing in OSA (Shi et al., 2017).

Based on the results of Li et al. (2013) and Shen et al. (2020), the SFG, a connecting node between the central executive network and default mode network (DMN), is linked to the cognitive control network and the cognitive executive network. The MFG, a location where the ventral and dorsal attention networks converge, is involved in language fluency, object naming and directed attention and is part of the cognitive executive network (Japee et al., 2015; Viñas-Guasch and Wu, 2017). The MTG has been proven to be an important structure in the memory consolidation process and a crucial node in the language network, as it participates in semantic control and social cognition (Acheson and Hagoort, 2013; Chen Y. et al., 2020). As one piece of evidence, we showed exaggerated connection weights from the left dAI to the bilateral SFG, right MFG and left PreCG and from the right dAI to the right MTG and left PreCG. Zhang et al. (2013) discovered impaired FC of the PreCG in OSA patients and confirmed structural and functional disruptions in the anterior DMN containing the SFG, and speculated these damages may be a probable candidate mechanism for cognitive and emotional disorders in patients with OSA. Furthermore, the increase in FC in the temporal gyrus of OSA patients has been associated with neurocognitive impairment, as affirmed by the study of Zhou et al. (2020). The brain areas with abnormal FC in our study overlapped with those previously reported to show structural or functional aberrations in OSA, such as the cerebellum, frontal lobe and temporal lobe, which are distributed in the cognitive control circuit along with the dAI and are responsible for memory, attention, language and executive control (Park et al., 2016a; Caporale et al., 2021). The exaggerated FC may bring about synaptic plasticity, which could alter the functional compensatory response in the early stages of cognitive impairments.

In addition, there was some degree of correlation between the MoCA score and FC of the left dAI to the right SFG (*t* = −2.051, *P* = 0.043), the left dAI to the left SFG (*t* = −2.344, *P* = 0.022), however, which only present before the Bonferroni correction. One possible explanation is that the OSA patients were in the early stage of neurological abnormalities, with mild cognitive symptoms and a small decrease in MoCA score, similar to the findings of several previous studies (Park et al., 2016a; Yu et al., 2019).

### Changed Functional Connectivity Between the Ventral Anterior Insula and Emotional Circuits

Patients with OSA often show emotional abnormalities, such as anxiety or depression (Kim et al., 2019). The vAI participates in the experience of negative emotions and shows abnormal activation in the studies of affect and emotion (Gogolla, 2017). In addition, a neuroimaging study revealed that the vAI is connected to the area representing the sensory input associated with emotional expression (Touroutoglou et al., 2012). Additionally, the connectivity of the vAI with other brain regions is altered in anxiety disorders (Grupe and Nitschke, 2013). We found altered FC between the vAI and regulatory networks of emotion including the CPL, superior parietal lobule, precuneus, superior/inferior temporal gyrus, fusiform gyrus, precentral and postcentral gyrus. The CPL is linked to the expression of cognitive sentiment, and emotional motor responses (Strata, 2015), with increased activation in the state of anxiety or depression (Pico-Perez et al., 2017), and its dysfunction has been observed in OSA (Yu et al., 2019). Bae et al. (2019) found that emotional perception test performance was associated with brain connectivity from the fusiform gyrus to the insula. Brain regions, incorporating the parietal lobe, frontal lobe, precuneus, temporal lobe and insula are activated in patients with depressive disorders or OSA patients with depression and anxiety symptoms (Pico-Perez et al., 2017; Song et al., 2018). Significantly, compared with those without emotional symptoms, OSA subjects with emotional symptoms have shown exaggerated damage in specific brain regions, including the insula, parietal lobe and temporal lobe (Cross et al., 2008; Kumar et al., 2009), which contain the emotion-related abnormal brain regions revealed in our results.

Reduced activation may reflect a defective response and dysfunction between the vAI and emotionally related areas of the bilateral CPL, superior parietal lobule, precuneus, and fusiform gyrus of OSA subjects. While, the exaggerated FC may bring about synaptic plasticity, which could alter the functional compensatory response in the early stages of mood disorders in OSA patients. It seems the left vAI plays a more important role than the right.

### Disturbed Functional Connectivity in the Posterior Insula and Sensorimotor Regions

In this study, disrupted FC was exhibited between the PI and PreCG, STG, MTG, SFG, medial frontal gyrus, and precuneus. Caused by upper respiratory tract muscle abnormalities in OSA, hypoxemia further disrupts sensorimotor function (Eckert et al., 2011). Previous studies have found structural and functional connectivity disorders in sensorimotor areas in patients with OSA. The PI is functionally connected with the motor, somatosensory and temporal cortices, which are associated with skeletal movement, body orientation, environmental monitoring and response selection, as demonstrated by FC research involving the insula (Cauda et al., 2011). In our study, significantly increased connection weights were observed between the bilateral PreCG, right MTG, STG and precuneus and the right PI, and increased connection weights were observed between the right STG, medial frontal gyrus, and left SFG and the left PI. The bilateral PreCG, namely, the premotor cortex, is part of one of the classic sensorimotor subregions in charge of complex motor task signal processing (Cooke and Graziano, 2004; Gao et al., 2020). Zhang et al. (2013) identified deficits in the sensorimotor-related brain networks associated with the PreCG and medial prefrontal cortex in patients with OSA as predictors of impaired motor function. Although the other abnormally connected brain regions are not in sensorimotor areas, they are important for different aspects of sensorimotor control. Neuroimaging data have shown that the temporal gyrus acts as a hub for action-related information and processing and participates in language control, linking semantic knowledge about meaningful action to sensorimotor activity (Rinat et al., 2020) and contributing to the kinesthetic perception of joint motion and higher-order sensory integration (Goossens et al., 2018). Abnormal blood-oxygen-level-dependent signals appear in the medial frontal gyrus and precuneus during hand and foot movement (Nakata et al., 2019). In addition, the correlation results indicated that the connection between the right precuneus and right PI was positively correlated with the AHI. Interestingly, an authoritative study demonstrated that the insular lobe acts as an intermediary in the sensorimotor-insular-frontal lobe loop, promoting the interaction between the primary sensorimotor cortex and higher regions (Namkung et al., 2017). Disruption of the FC in the sensorimotor area-posterior insula in OSA may signify abnormal sensorimotor activity, deserving our attention.

### Impaired Functional Connectivity in the Cerebellum and Default Mode Network

Surprisingly, our results revealed that all insular subregions had impaired connections with the cerebellum. Several neuroimaging results have also supported the idea that the cerebellum is the most structurally and functionally affected area in OSA subjects (Macey et al., 2008; Kumar et al., 2012; Park et al., 2016b). The cerebellum is regarded as having a considerable border role in sensory and perceptual processing (Baumann et al., 2015) that is not only germane to motor function (Goossens et al., 2018) but also related to cognitive processing and emotional control (Moreno-Rius, 2018). Lobule VI is part of the neural circuit of fear and anxiety (Lange et al., 2015). In addition, the cerebellum is involved in maintaining sleep, a lack of which may disrupt cerebellar function (Shi et al., 2017). Whether cerebellar abnormalities can truly explain the clinical symptoms of OSA from the perspective of neuroimaging requires further exploration, and could possibly be the focus of our next study. A recent study about cerebellar functional changes in OSA enhanced our confidence (Park et al., 2021).

The right AI is the critical node of the salience network (Touroutoglou et al., 2016), and serves to switch between the central executive network and DMN (Sridharan et al., 2008). A study of functional separation of the right AI revealed that OSA selectively damaged the rs-FC between the AI and the DMN, which may be a substrate for cognitive impairment in OSA patients (Zhang et al., 2015). The DMN is composed of two spatially independent subnetworks: the anterior DMN and the posterior DMN. The anterior DMN contains the SFG and is mainly responsible for emotion self-reference and management; however, the posterior DMN contains the precuneus and primarily presides over cognitive processing and memory extraction (Coutinho et al., 2016). Previous studies concluded that depression and anxiety were associated with DMN alterations, which highlights the role of the DMN in emotional processing (Vicentini et al., 2017). Our results of FC between the left vAI and posterior DMN was decreased, while FC between the left dAI and anterior DMN showed increased suggested a discrete connectivity between the AI and anterior/posterior DMN, consistent with the disruption of the DMN in OSA patients (Chen et al., 2018).

### Limitations

Several limitations should be noted in our study. First, we recruited only adult men with OSA, given the difficulty of sample collection and convention of homogeneity. Women and children with OSA need to be included in future studies. Second, the evaluation of the emotional state was not included in this study, since only a small number of patients were carried out with emotional assessments. Fortunately, previous studies have reported emotional abnormalities in OSA patients. An emotion scale should be added to better verify our findings in future studies. Finally, external physiological recordings were not obtained during fMRI acquisition (e.g., respiratory belt and photoplethysmography). Several recent studies have indicated that fluctuations in physiological processes may lead to artificial FC (Bright et al., 2020; Chen J. E. et al., 2020; Xifra-Porxas et al., 2021). Physiological recordings could in principle help to address whether the differences in insular subregions connectivities are related to neuronal brain activity or physiological fluctuations and should be included in future studies.

### Conclusion

In summary, the insular subregions showed extensive abnormal connectivities with other brain areas in OSA patients. These abnormal connectivities were manifested in disrupted connections from the vAI to emotional regions, from the dAI to cognitive regions, from the PI to sensorimotor and language regions, and from the AI to DMN regions, where there exist in partial functional overlap rather than complete independence. This disorganized FC associated with neuropsychological disorders, may be responsible for the evoked sensorimotor, affective, and ventilatory challenges and cognitive deficits in OSA.

## Data Availability Statement

The datasets presented in this article are not readily available because these data are related to the follow-up longitudinal study of our research group, and the datasets are still being replenished. Requests to access the datasets should be directed to LK, dockong666@163.com.

## Ethics Statement

The studies involving human participants were reviewed and approved by Ethics Committee of The First Affiliated Hospital of Nanchang University. The patients/participants provided their written informed consent to participate in this study.

## Author Contributions

DP guided and designed the MRI experiment. HL analyzed the resting-state fMRI data. LK and YS analyzed and discussed the ideas of the manuscript. LK organized the results and wrote the manuscript. LK, XL, PL, KL, WX, and YZ collected the resting fMRI data and applied for ethics approval. HL and DP reviewed and revised the manuscript. All authors contributed to the article and approved the submitted version.

## Conflict of Interest

The authors declare that the research was conducted in the absence of any commercial or financial relationships that could be construed as a potential conflict of interest.

## Publisher’s Note

All claims expressed in this article are solely those of the authors and do not necessarily represent those of their affiliated organizations, or those of the publisher, the editors and the reviewers. Any product that may be evaluated in this article, or claim that may be made by its manufacturer, is not guaranteed or endorsed by the publisher.

## Footnotes

1
www.MRIcro.com

2
http://rfmri.org/dpabi

3
http://afni.nimh.nih.gov/afni/

4
http://restfmri.net/forum/REST_V1.8

5
http://www.alivelearn.net/xjview
